# Temporomandibular disorders among adult patients: Relationship with personality traits and other factors

**DOI:** 10.1371/journal.pone.0345159

**Published:** 2026-03-23

**Authors:** Hassan Adnan Alshawaf, Mohammed Ali Alnemer, Majid Alawi Alsafwani, Faisal Abdulmonem Alhalal, Moayad Mohammed Aljeshi, Muhammad Ashraf Nazir

**Affiliations:** 1 College of Dentistry, Imam Abdulrahman Bin Faisal University, Dammam, Saudi Arabia; 2 Department of Preventive Dental Science, College of Dentistry, Imam Abdulrahman Bin Faisal University, Dammam, Saudi Arabia; University of North Carolina at Chapel Hill Davis Library: The University of North Carolina at Chapel Hill Libraries, UNITED STATES OF AMERICA

## Abstract

**Introduction:**

Temporomandibular disorders (TMDs) refer to the pain and dysfunction of the temporomandibular joints and associated masticatory muscles. There is a lack of evidence about the relationship between TMDs and personality traits. The aim of the study was to evaluate temporo-mandibular disorders and their relationship with personality traits and other study variables among adult patients in Dammam, Saudi Arabia.

**Methods:**

This cross-sectional study was conducted at the Dental Hospital of the College of Dentistry, Imam Abdulrahman Bin Faisal University (IAU), Dammam, Saudi Arabia. Adult male and female patients attending dental hospital who provided written consent were included in the study. A self-administered questionnaire was administered among participants, which included demographic information, Big-Five Inventory-2 Short form (BFI-2 S) for personality traits, and Fonseca’s questionnaire for temporo-mandibular disorders (TMDs). A t-test, one-way ANOVA test, and multiple linear regression analysis were performed in the study.

**Results:**

The study included 500 participants, with 66.4% males and 33.6% females. Most participants (59.2%) had TMDs, with 40% mild TMDs, 14.6% moderate TMDs, and 4.6% severe TMDs. Females demonstrated a significantly higher mean score of Fonseca’s questionnaire (31.79 ± 21.96) compared to males (22.32 ± 18.75) (P < 0.001). The participants with no education had the highest mean score of Fonseca’s questionnaire (52.50 ± 33.06) compared to those with school education (27.09 ± 18.96) and college/university education (23.49 ± 19.78) (P < 0.001). The participants with arthritis and sleep disorders demonstrated significantly greater severity of TMDs than those without these conditions (P < 0.001). There was a significant negative correlation between TMDs and agreeableness (r = −0.26, P < 0.001) and conscientiousness (r = −0.23, P < 0.001). However, a significant positive correlation (r = 0.33, P < 0.001) can be observed between TMDs and neuroticism. According to multiple linear regression analysis models, agreeableness (B = −1.17, P < 0.001), conscientiousness (B = −1.04, P < 0.001), and neuroticism (B = 1.45, P < 0.001) remained statistically significant predictors of TMDs after controlling for age, gender, nationality, education, and monthly income.

**Conclusion:**

The study found that TMDs were highly prevalent among adult patients. TMDs were significantly related to female gender and low education level. The participants with arthritis and sleep disorders demonstrated significantly increased severity of TMDs. Neuroticism was significantly correlated with the severity of TMDs. On the other hand, agreeableness and conscientiousness were negatively correlated with TMDs and emerged as protective predictors against TMDs. Adult patients should be screened for TMDs and personality traits, and multidisciplinary treatment plans involving treatments for TMDs and psychological support should be tailored for them.

## Introduction

The personality of an individual consists of relatively stable patterns of thoughts, feelings, and behaviors along with underlying psychological mechanisms [1]. The Big Five or Five Factor Model classifies personality into five categories, such as agreeableness, neuroticism, extraversion, conscientiousness, and openness to experiences [2]. This model has been widely used with different measurement methods among individuals of different ages and has demonstrated cross-cultural validity [3]. Personality traits may affect an individual’s dental perceptions as well as satisfaction with dentition and are associated with parafunctional habits, oral hygiene, and dental caries [4–6]. Additionally, neuroticism is known to significantly compromise oral health-related quality of life, while extraversion improves it [7].

Temporomandibular disorders (TMDs) refer to the pain and dysfunction of the temporomandibular joints and associated masticatory muscles, affecting 31.1% adult and elderly populations globally [8,9]. In Saudi Arabia, about 40% of the population is affected with TMDs, and higher prevalence is found in females than males [10]. Moayedi et al. observed that the duration of TMDs contributed to abnormalities in the gray matter within brain areas of patients with chronic painful TMDs and neuroticism was also positively correlated with brain changes [11]. In Brazil, Serra-Negra et al. found that sleep bruxism was common among participants with high score of neuroticism [12]. Southwell et al. showed that patients with TMDs demonstrated significantly greater neuroticism and introversion compared to those without TMDs in the U.K [13]. Similarly, a recent study in Poland reported that personality traits were significantly associated with TMDs [14].

There is a paucity of comprehensive data evaluating the personality profile of dental patients with TMDs, making it difficult to ascertain if certain personality traits increase the likelihood of TMDs among them. The study may enhance health professionals’ understanding of the interrelationship between personality and TMDs and play their role in the early diagnosis and prevention of TMD. Therefore, there was a need to conduct a study to investigate personality traits of patients and their association with TMDs. The aim of this study was to evaluate temporo-mandibular disorders and their relationship with personality traits and other factors among adult patients in Dammam, Saudi Arabia. It was hypothesized that specific personality traits are correlated with the severity of TMDs among dental patients.

## Materials and methods

### Study design and population

A cross-sectional questionnaire-based study was conducted at the Dental Hospital of the College of Dentistry, Imam Abdulrahman Bin Faisal University (IAU). The Institutional Review Board at the Deanship of Scientific Research IAU approved the study (IRB-2025-02-0055). Adult male and female patients attending dental hospital who provide written consent were included in the study. Patients with arthritis were included in the study to evaluate relationship between arthritis and TMDs severity. Children and adolescents seeking dental care at the dental hospital, patients outside the IAU dental hospital, patients who refused to sign consent, and patients who do not understand English or Arabic language due to language barriers were excluded from the study. In addition, patients with uncontrolled diabetes, severe cardiovascular, liver, and kidney diseases, psychological conditions, and those seeking treatment for TMDs were excluded from the study. A 95% confidence level, 0.5 population variability, ± 5% margin of error, and an approximate population (N ≈ 10,000) were used to compute the sample size of 554 participants. The study employed a convenience sampling technique for data collection. The researchers started data collection on 23 January 2025 and completed on 27 March 2025.

### Study instrument

A self-administered questionnaire consisted of three sections. The first section included demographic information, the second section consisted of the Big-Five Inventory-2 Short form (BFI-2 S), and the third section contained Fonseca’s questionnaire. The Big-Five Inventory-2 Short form (BFI-2 S) was used to evaluate personality traits of the participants. The scale has excellent reliability and validity and has been validated in various languages, including Arabic language [15,16]. It consists of 30 items, which describe the major personality traits such as extraversion (6 items), agreeableness (6 items), conscientiousness (6 items), negative emotionality/neuroticism (6 items), and openness (6 items). Each item in the scale uses a 5-point Likert scale ranging from strongly disagree to strongly agree [16].

The Fonseca’s questionnaire is used to collect epidemiological information regarding the prevalence and severity of TMDs [17]. The scale has ten questions: five assess the patient’s emotional status and how they perceive their TMDs, and the remaining five questions evaluate pain in the head, back, or temporomandibular joint, or during chewing, as well as parafunctional behaviors, restricted movement, and joint clicking. The potential responses to each question are “yes” (10 points), “no” (0 points), and “sometimes” (5 points). The total score is calculated by adding individual item scores, and the maximum score is 100. TMDs are categorized as mild when a total score is between 20 and 40, moderate when it is between 45 and 65, severe when it is 70–100, and absent when it is less than 16 [17,18]. Arabic and non-Arabic speaking populations responded to Arabic and non-Arabic versions of the Fonseca questionnaire [19].

### Procedure and ethics

The participants were invited to complete questionnaires in person. The researchers distributed questionnaires among participants in the waiting area before dental treatment in the dental hospital. Hard copies of the questionnaire were administered among study participants, and completed questionnaires were returned to the researchers. The participants received information about the research project, including its aim and potential advantages. They were assured of their voluntary participation in the study and the confidentiality and privacy of their responses. Explanations or clarifications were provided to those participants who had difficulty understanding any items in the questionnaire.

### Data analysis

The Statistical Package for the Social Sciences (IBM SPSS Statistics for Windows, version 22.0. Armonk, NY: IBM Corp) was used for statistical analysis. T-test and one-way ANOVA test were performed to determine the relationship between TMDs and demographic and other variables. The relationship of personality traits and TMDs was explored by using Pearson’s correlation test and multiple linear regression analysis. MANCOVA analysis was performed to determine whether personality traits differ between TMDs categories. A *P* < 0.05 was used for statistical significance.

## Results

The study included 500 participants, with 66.4% males and 33.6% females. Most participants were Saudis (93%), aged 18–49 years (77.2%), had college/university education (61.2%), and belonged to the middle-income group (54.2%). Females demonstrated a significantly higher mean score of Fonseca’s questionnaire (31.79 ± 21.96) compared to males (22.32 ± 18.75) (P < 0.001). The participants with no education had the highest mean score of Fonseca’s questionnaire (52.50 ± 33.06) compared to those with school education (27.09 ± 18.96) and university education (23.49 ± 19.78) (P < 0.001). The participants with arthritis, bruxism, and sleep disorders demonstrated significantly higher TMDs than those without these conditions (<0.001) (Table 1).

**Table 1:** Relationship between TMD and demographic and other factors among study participants.

Study variables	N (%)	Fonseca’s questionnaire score Mean±SD	P-value
**Age**			0.479
18-49 years	386 (77.2)	26.10 ± 20.90	
50-64 years	73 (14.6)	23.48 ± 19.40	
≥65 years	41 (8.2)	23.44 ± 16.61	
**Gender**			<0.001
Male	332 (66.4)	22.32 ± 18.75	
Female	168 (33.6)	31.79 ± 21.96	
**Nationality:**			0.426
Saudi	465 (93.0)	25.30 ± 19.98	
Non-Saudi	35 (7.0)	28.14 ± 25.01	
**Level of education:**			<0.001
No education.	12 (2.4)	52.50 ± 33.06	
School education	182 (36.4)	27.09 ± 18.96	
College/University education	306 (61.2)	23.49 ± 19.78	
**Monthly family income:**			0.534
Low:	149 (29.8)	24.11 ± 16.31	
Middle	271 (54.2)	26.39 ± 21.01	
High	80 (16.0)	25.06 ± 24.59	
**Arthritis**			<0.001
Yes	80 (16.0)	34.19 ± 22.70	
No	420 (84.0)	23.84 ± 19.48	
**Sleep disorders**			<0.001
Yes	79 (15.8)	35.0 ± 22.35	
No	421 (84.2)	22.91 ± 19.0	

**Fig 1:**
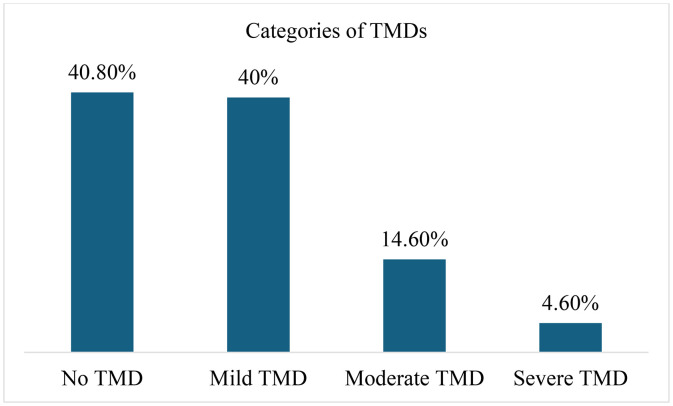
Distribution of TMDs among study participants.

Fig 1 shows TMDs categories: 40.8% of the participants had no TMDs, 40% mild TMDs, 14.6% moderate TMDs, and 4.6% severe TMDs.

**Fig 2:**
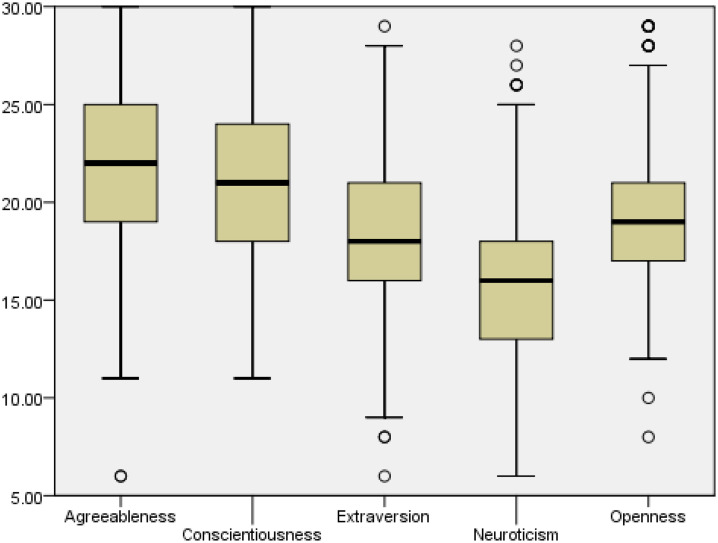
Boxplots of BFI-2-S personality traits scores.

Fig 2 show boxplots of the BFI-2-S personality traits scores among study participants.

The median scores of agreeableness and conscientiousness were greater compared to other traits. On the other hand, neuroticism showed the lowest median score among personality traits.

Table 2 shows that there was a significant negative correlation between agreeableness and TMDs (r = −0.26, P < 0.001). A similar significant and negative correlation was found between conscientiousness and TMDs (r = −0.23, P < 0.001). However, a significant positive correlation (r = 0.33, P < 0.001) was observed between TMDs and neuroticism.

**Table 2:** Bivariate analysis: Relationship between TMD and personality traits among study participants.

Variables	Correlation coefficient	P-value
**TMDs and Agreeableness**	−0.26	<0.001
**TMDs and Openness**	−0.08	0.077
**TMDs and Neuroticism**	0.33	<0.001
**TMDs and Extraversion**	−0.08	0.062
**TMDs and Conscientiousness**	−0.23	<0.001

According to multiple linear regression analysis models, agreeableness (B = −1.17, P < 0.001), conscientiousness (B = −1.04, P < 0.001) and neuroticism (B = 1.45, P < 0.001) remained statistically significant predictors of TMDs after controlling for age, gender, nationality, education, and monthly income. (Table 3). These models showed normal P-P plots with regression standardized residuals normally distributed along a diagonal line, satisfying the assumption of normality. The tolerance (0.87–0.99) and Variance Inflation Factor (VIF) (1.01–1.16) values revealed that independent variables in these models did not significantly correlate with each others, suggesting no issue of multicollinearity.

**Table 3:** Multivariable analysis: Relationship between TMDs and personality traits among study participants.

Study variables	Unstandardized coefficients		P-value	95.0% Confidence interval for B
	B	Std. Error		
**Neuroticism**	1.45	0.20	<0.001	(1.06, 1.84)
**Conscientiousness**	−1.04	0.20	<0.001	(−1.44, −0.63)
**Agreeableness**	−1.17	0.19	<0.001	(−1.55, −0.78)

The regression model assessing the relationship between neuroticism and TMDs explained 17.2% of the variance in TMD scores (R² = 0.172). Similarly, the models for conscientiousness and TMDs and agreeableness and TMDs explained 12.8% (R² = 0.128) and 14.5% (R² = 0.145) of the variance in TMD scores, respectively.

The results showed statistically significant relationships between TMDs categories and agreeableness (p < 0.001) and conscientiousness (p < 0.001). As the mean scores of these traits increase, the severity of TMDs decreases. On the other hand, an increase in the mean score of neuroticisms was significantly related to an increase in TMDs severity (p < 0.001) (Table 4).

**Table 4:** Estimated marginal means of personality traits by TMD categories among study participants.

Personality traits	TMD categories	Estimated marginal mean	Std. Error	95% Confidence Interval (CI)	P-value
Extraversion	No TMDs	18.65	0.24	(18.19, 19.12)	0.255
	Mild TMDs	18.27	0.24	(17.80, 18.73)	
	Moderate TMDs	17.87	0.39	(17.09, 18.64)	
	Severe TMDs	17.67	0.71	(16.28, 19.06)	
Agreeableness	No TMDs	23.33	0.30	(22.75, 23.92)	<0.001
	Mild TMDs	22.23	0.30	(21.64, 22.82)	
	Moderate TMDs	20.07	0.50	(19.09, 21.04)	
	Severe TMDs	19.62	0.90	(17.87, 21.38)	
Conscientiousness	No TMDs	22.91	0.29	(22.34, 23.47)	<0.001
	Mild TMDs	20.67	0.29	(20.11, 21.24)	
	Moderate TMDs	20.12	0.48	(19.18, 21.06)	
	Severe TMDs	20.37	0.86	(18.68, 22.06)	
Neuroticism	No TMDs	13.92	0.28	(13.37, 14.48)	<0.001
	Mild TMDs	16.36	0.28	(15.80, 16.92)	
	Moderate TMDs	16.12	0.47	(15.20, 17.05)	
	Severe TMDs	19.53	0.85	(17.87, 21.20)	
Openness	No TMDs	19.62	0.24	(19.15, 20.09)	0.254
	Mild TMDs	19.76	0.24	(19.29, 20.24)	
	Moderate TMDs	18.90	0.40	(18.11, 19.68)	
	Severe TMDs	18.97	0.72	(17.56, 20.38)	

Covariates: age, gender, education, nationality, and monthly family income

## Discussion

The present study evaluated the prevalence of TMDs and their relationships with personality traits and other factors among adult patients in Dammam, Saudi Arabia. The study found a high prevalence of TMDs in our sample of patients with 40% having mild TMDs, 14.6% moderate TMDs, and 4.6% severe TMDs. In a similar recent study from Saudi Arabia that utilized the Fonseca questionnaire, mild TMDs were observed in 41.4%, moderate TMDs in 20.5%, and severe TMDs in 11.4% of adult participants [20]. Likewise, a higher prevalence of TMDs (68.5%) was reported in another study of adults from Saudi Arabia, and 41.5% of participants demonstrated mild TMDs, 19.3% moderate TMDs, and 7.8% severe TMDs [21]. Another previous study also administered Fonseca’s questionnaire among adult patients and observed TMDs in 61% of the study population in Madinah, Saudi Arabia [22]. Stress, anxiety, depression, sleep problems, parafunctional habits, and cultural and societal influences may account for a high prevalence of TMDs among adults in the country [10].

Nadershah reported that a significantly greater proportion of females (42%) compared with males (28%) demonstrated TMDs in Jeddah, Saudi Arabia [23]. Likewise, a study by Alolayan et al. showed that TMDs affected more females (55.7%) than males (44.3%) in Madinah, Saudi Arabia [22]. In a recent study, Pinheiro and his coworkers confirmed that Brazilian women were significantly more likely (odds ratio = 1.91) than men to experience the symptoms of TMDs [24]. In a study of the adult Finnish population by Qvintus et al., females were shown to have a greater risk for TMD problems compared with males [25]. Females were also shown to demonstrate greater disability and psychosocial impairment due to TMDs pain [26]. In the present study, females reported experiencing significantly greater severity of TMDs than their male counterparts. Greater prevalence and severity of TMDs among women than men can be attributed to the difference in their pain perception, pain coping strategies, psychological factors, hormonal influences, or sociocultural norms, including oral care seeking behaviors [24].

In the present study, the participants with no education reported significantly higher severity of TMDs than those with school or college/university education. This agrees with the findings of Banafa et al., who reported that pain-related TMD signs in adults were significantly related to their low education [27]. Similarly, Qvintus et al. showed that participants with low education were significantly more likely to demonstrate signs and symptoms of TMDs than those with high education [25]. Individuals with lower educational attainment may experience more anxiety and depressive disorders, and social and economic challenges, which may predispose them to a greater burden of TMDs than those with higher levels of education [25,26].

The present study observed a significantly greater severity of TMDs among participants with arthritis and sleep disorders than those without these conditions. The literature suggests that inflammatory biomarkers of rheumatoid arthritis are known to increase the progression of TMDs, and the greater the duration of rheumatoid arthritis, greater the severity of TMDs [28]. Additionally, rheumatoid arthritis is known to cause changes in the bone structures of temporomandibular joint, such as erosion, sclerosis, flattening, and osteophytes [29]. There is a bilateral relationship between sleep disorders and TMDs, and patients with TMDs may experience sleep disorders, and individuals with sleep disorders may develop TMDs [30,31]. It was reported that the participants with poor sleep quality were 2.89 times more likely to develop TMDs [31]. In Taiwan, a national cohort study showed that patients with sleep apnea demonstrated significantly higher incidence of TMDs than controls, and sleep apnea was identified as an independent risk factor for the development of TMDs [30].

Neuroticism is associated with an increased vulnerability to poor oral hygiene and gingival health and low oral health related quality of life [7,32]. Evidence from a neurobiological study suggests that neuroticism may affect the function and structure of the brain, contributing to the pathophysiology of TMDs [11]. Likewise, it was reported that high neuroticism scores were associated with more disability days among patients with painful TMDs [26]. Similarly, the participants with symptoms of TMD were shown to demonstrate higher scores of neuroticism than those without TMD symptoms [14]. In our sample of adult patients, neuroticism was significantly correlated with TMDs severity after controlling for demographic variables, suggesting higher scores of neuroticism may result in greater severity of TMDs. It is known that the patients with higher scores of neuroticisms experience heightened distress and low tolerance to pain, hence, they are more likely to perceive signs and symptoms of TMDs [11].

According to the Big Five Model, individuals with high scores in conscientiousness personality tend to be diligent, careful, organized, goal oriented, and self-disciplined and exhibit careful planning, persistence in pursuing objectives, and achievement of goals [33]. Evidence suggests that conscientiousness is associated with lower biomarkers of inflammation and cardiovascular malfunction, suggesting improved health outcomes among individuals with high conscientiousness [34]. It is also known that people with high conscientiousness demonstrate improved oral health behaviors such as toothbrushing, oral hygiene practices, dental attendance, and oral health awareness [6]. In a study of the adult population in Saudi Arabia, Almutairi et al., showed a significantly lower prevalence of TMDs among participants with conscientiousness [35]. Similarly, Mitrowska-Guźmińska et al., reported that patients with pain related TMDs exhibited a significantly lower level of conscientiousness compared to those without TMD pain symptoms [14]. In line with existing literature, higher scores of conscientiousness were significantly and negatively correlated with severity of TMDs in our sample of patients.

People high in agreeableness are cooperative, trusting, and sympathetic, and they establish stronger social support networks and cope better with stresses [33]. Almutairi et al., and Mitrowska-Guźmińska et al. observed no significant association between agreeableness and TMDs [14,35]. However, the regression analysis in the present study confirmed a significant and negative correlation between agreeableness and TMDs, suggesting agreeableness as a protective factor against TMDs. Significantly reduced severity of TMDs related to agreeableness in the present study could be explained by the existence of coping abilities and social support networks in people high in agreeableness, resulting in reduction of stress and anxiety, which are main predictors of TMDs [26]. Significant interrelationships between neuroticism, conscientiousness, agreeableness and TMDs highlight the importance of evaluating personality traits in TMDs patients to inform comprehensive management strategies.

A large sample of adult patients was one of the strengths of this study. In addition, the study used validated instruments to screen patients with TMDs and evaluate their personality traits. The study added useful information to the limited current literature on the relationship between TMDs and personality traits in adult populations. However, there are certain limitations to this study. First, limited generalizability of results to adult populations in other geographic locations due to data collection from an institution. Second, under and over reporting in self-reported surveys can lead to measurement bias in the study. Third, a cross-sectional study design is limited in establishing temporal relationships between study variables because data are collected at the same time. Despite these limitations, a cross-sectional study design is suitable for screening patients with TMDs, evaluating personality traits, and exploring the relationships between personality traits and TMDs. In addition, a cross-sectional study is time-efficient and less resource-consuming, providing valuable information for the generation of hypotheses for future cohort studies. Therefore, a multicenter cohort study should evaluate the interrelationships between TMDs and personality traits and other variables by including clinical diagnoses of TMDs from populations with diverse cultural backgrounds.

## Conclusions

The study found that TMDs were highly prevalent among adult patients. TMDs were significantly related to female gender and low education level. The participants with arthritis and sleep disorders demonstrated significantly increased severity of TMDs. Neuroticism was significantly correlated with the severity of TMDs. On the other hand, agreeableness and conscientiousness were negatively correlated with TMDs, and these emerged as protective factors against TMDs. The study underscores the importance of personality traits for a more holistic approach in the prevention and management of patients with TMDs. The decision makers should develop policies and procedures to screen dental patients for TMDs and personality traits. Comprehensive multidisciplinary approaches, including therapeutic interventions for TMDs, psychological support or stress management, should be tailored especially for patients with severe TMDs and neuroticism.

## Supporting information

S1 File Data on personality traits and TMD. (XLS)
